# Women Have a Preference for Their Male Partner to Be HPV Vaccinated

**DOI:** 10.1371/journal.pone.0097119

**Published:** 2014-05-14

**Authors:** Diane Medved Harper, Natalie Marya Alexander, Debra Ann Ahern, Johanna Claire Comes, Melissa Smith Smith, Melinda Ann Heutinck, Sandra Martin Handley

**Affiliations:** 1 Department of Biomedical and Health Informatics, University of Missouri Kansas City School of Medicine, Kansas City, Missouri, United States of America; 2 Department of Community and Family Medicine, University of Missouri Kansas City School of Medicine, Kansas City, Missouri, United States of America; 3 Department of Obstetrics and Gynecology, University of Missouri Kansas City School of Medicine, Kansas City, Missouri, United States of America; 4 Department of Family Medicine, Kansas City University of Medicine and Biosciences, Kansas City, Missouri, United States of America; 5 University of Missouri Kansas City Student Health and Wellness, University of Missouri-Kansas City, Kansas City, Missouri, United States of America; IPO, Inst Port Oncology, Portugal

## Abstract

**Background:**

Peer influence and social networking can change female adolescent and young adult behavior. Peer influence on preferences for male human papillomavirus (HPV) vaccination has not been documented. The primary aim of this study was to determine if women had preferences about male sexual partner HPV vaccination receipt.

**Methods and Findings:**

A prospective survey of women 18–26 years of age was conducted at an urban university student health clinic. Education about the two HPV vaccines, cervical cancer and genital warts was provided. Women self-reported their demographic and medical history data, as well as their own preferences for HPV vaccine and their preferences for their male partner HPV vaccine using a 5 point Likert scale. 601 women, mean age of 21.5 years (SD 2.4), participated between 2011 and 2012. Nearly 95% of respondents were heterosexual; condoms and contraceptives were used in over half of the population. Regardless of the woman's vaccination status, women had significantly higher (strongly agree/agree) preferences for the male partner being vaccinated with HPV4 than not caring if he was vaccinated (63.6% vs. 13.1%, p<0.001). This preference was repeated for sexual risk factors and past reproductive medical history. Women who received HPV4 compared to those choosing HPV2 had a significantly lower proportion of preferences for not caring if the male partner was vaccinated (13% vs. 22%, p = 0.015).

**Conclusions:**

Women preferred a HPV vaccinated male partner. Peer messaging might change the male HPV vaccination uptake.

## Introduction

Preferences for prophylactic HPV vaccines have been studied from the perspective of parents [1]–[4], health care providers [5], [6], and the individual adolescent/college aged female [7]–[9] and male [10], [11]. These works illuminate attitudes and reasons for accepting or rejecting HPV vaccination. The most powerful motivator for young adult behavior, though, is one that is peer-induced [12]–[15] or from within a social network [16], [17]. No formal work to date has elicited female preferences for male partner vaccination in the context of HPV vaccination. This pairing is unique in that only one vaccine is approved for use in males, and that vaccine has considerably more evidence for genital wart efficacy than anal intraepithelial neoplasia grade 3 (AIN 3) or cancer efficacy for the male population [18]. For women, while there are two approved HPV vaccines, practice patterns in countries tend to be skewed towards offering only one, whether due to direct-to-consumer advertising or due to national tenders. In the United States, HPV4 has the majority market share for female vaccination. Understanding women's preferences for male partner HPV status has not been explored.

The primary aim of this study was to determine the preference rankings women have about HPV vaccination for a male sexual partner and how these vary by her reproductive health experiences.

## Methods

This prospective study was approved by the University of Missouri Kansas City (UMKC) Social Sciences Adult Institutional Review Board (SSIRB) (#SS10-56X) as an exempt study not requiring verbal or written consent and is part of a 601 person study on how women decide to accept or reject the HPV vaccination [19].

All women under 27 years old presenting for care at the UMKC Student Health and Wellness office between January 2011 and August 2012 were invited at the time of check-in to participate in this cervical cancer prevention educational intervention. This time frame allowed for 5 academic terms to be surveyed providing the opportunity to reach as many female students as possible, including those who may have taken time away.

Cervical cancer prevention information was written at the seventh grade reading level, as required by the SSIRB. It addressed the three methods of prevention in this age group: Pap testing, each of the two prophylactic HPV vaccines and the combination of Pap testing and HPV vaccination. The strengths and limitations of the three methods were outlined including colposcopy and treatment of detected abnormalities, the HPV genotypes directly covered by each vaccine as well as those prevented by cross-protection, the efficacy of less than three doses, and current evidence for duration of vaccine efficacy. Genital wart coverage was included. Subjects self-reported demographic and past medical history information.

The HPV vaccine preference survey used a 5 point Likert scale (1 = strongly disagree to 5 = strongly agree) to elicit women's preferences for her male sexual partner. Partner preference choices were presented as “I want my sexual partner to be vaccinated with Gardasil to prevent spreading warts to me” vs. “I don't care if my sexual partner is vaccinated”. If she did not have a male partner, her survey was censored. We did not query preferences for concurrent male partners. No identifying information was collected.

### Statistics

Frequencies, Chi-square and Fisher's exact test were used to show nonrandom associations between two categorical variables with a p<0.05 considered significant. Mann Whitney U testing was used to compare median preference rankings; Students' t-test was used for simplifying reporting. Statistica version 12 was used for the analysis [20]. Data pertaining to cervical cancer screening experiences were limited to those age eligible women 21 years and older.

## Results

Over the five academic terms from January, 2011 through August 2012, 601 women under the age of 27 years agreed to participate in the cervical cancer prevention educational intervention. The average age of the participating woman was 21.5 years (SD 2.4), of whom 62% were white, 18% black, 4% Hispanic, 9% Asian and 7% of other races/ethnicities (Table 1). Over 90% had not been pregnant nor had a child. The median age at first intercourse was 17 years old, and the median number of sexual partners was 3. Sexual preferences were 94% heterosexual. Condoms and oral contraceptives were used either currently or ever in over half of the population. Of the age-eligible women 21 years and older, 85% had a Pap test in the past, 24% had an abnormal Pap and 15% had been to colposcopy; 12% knew they had had an HPV infection.

**Table 1:** Demographics of Student Population.

	N = 601	mean (SD)
**Age, years**	590	21.5 (2.4)
**Race**	**n**	**%**
White	378	62.3
Black	110	18.2
Hispanic	24	4.0
Asian	53	8.7
Other	41	6.8
**Gravidity**		
n = 0	506	90.2
n≥1	55	9.8
**Parity**		
n = 0	540	96.4
n≥1	20	3.6
**Educational Level**		
Undergraduate	393	66.7
Graduate	111	18.8
Professional (MD, JD)	85	13.8
BA/MS Program	4	0.7
**Income**		
<$10,000	212	37.1
$10,000–$30,000	168	29.4
$30,000–$60,000	93	16.3
>$60,000	98	17.2
**Age at First Intercourse, years**		
<14 years	11	2.2
14–15 years	74	15.2
16–17 years	200	41.2
18–20 years	171	35.2
>20 years	30	6.2
**Number of Lifetime sexual partners**		
0	65	12.0
1–2	204	37.5
3–5	159	29.2
6–10	80	14.7
>10	36	6.6
**Current Relationship Status**		
Single	503	85.0
Married	39	6.6
Divorced/Widowed	5	0.8
Partnered	45	7.6
**Contraceptive Use**		
Current/Ever	311	52.8
Never	278	47.2
**Condom Use**		
Yes	347	61.0
No	216	38.0
Sometimes	6	1.0
**History of Pap Screening** a		
Yes	303	85.4
No	52	14.6
**History of Abnormal Pap Screening** a		
Yes	86	24.1
No	271	75.9
**History of HPV Infection** a		
Yes	41	11.5
No	316	88.5
**History of Colposcopy** a		
Yes	52	14.6
No	305	85.4
**History of Genital Warts**		
Yes	15	2.6
No	574	97.4
**History of STI other than HPV**		
Yes	62	10.5
No	528	89.5
**Sexual Preferences**		
Only Men	544	94.4
Only Women	12	2.1
Men and Women	20	3.5

a among women 21 years and older.

Overall, women had significantly higher preferences for a male partner who was vaccinated than not (median 4 (95% CI: 3–5) vs. 2 (1–3); mean rank 3.9 (SD 1.1) vs. 2.4 (1.1), p<0.001). Table 2 shows that about a quarter of women, regardless of her vaccination status, were neutral in their preference ranking for male partner HPV vaccination. Women not already vaccinated and choosing HPV2 had a significantly lower proportion of high rankings (agree/strongly agree, Likert score of 4/5) for male partner vaccination than women who had at least one dose of prior HPV4 vaccination (67% vs. 75%, p = 0.049); while at the same time having a greater proportion of high rankings for not caring if the male partner was vaccinated (22% vs. 13%, p = 0.015).

**Table 2:** Women's preference rankings for her male partner's vaccination status.

	Strongly disagree/disagree			Neutral			Strongly agree/agree		
	n	%	p-value	n	%	p-value	n	%	p-value
Not vaccinated and would choose HPV2 for herself									
Male sexual partner vaccinated with HPV4	15	8.4%	**<0.0001**	44	24.7%	NS	119	66.9%*	**<0.0001**
Don't care if male partner is vaccinated	86	49.4%		50	28.7%		38	21.9%†	
Not vaccinated and would choose HPV4 for herself									
Male sexual partner vaccinated with HPV4	3	3.3%	**<0.0001**	21	22.8%	NS	68	73.9%	**<0.0001**
Don't care if male partner is vaccinated	53	57.6%		27	29.3%		12	13.1%	
Already vaccinated									
Male sexual partner vaccinated with HPV4	23	8.3%	**<0.0001**	45	16.3%	NS	208	75.4%*	**<0.0001**
Don't care if male partner is vaccinated	176	64.0%		63	22.9%		36	13.1%†	

*Women not already vaccinated and choosing HPV2 had a significantly lower proportion of strongly agree/agree rankings regarding having her male partner be vaccinated with HPV4 than do vaccinated women (70% vs. 75%, p = 0.049).

† Women not already vaccinated and choosing HPV2 had a significantly higher proportion of strongly agree/agree rankings regarding having her male partner be vaccinated with HPV4 than do vaccinated women (22% vs. 13%, p = 0.015).

Table 3 extends the preference rankings for male partner vaccination among those women with at least one dose of prior HPV4 vaccination and those women choosing HPV2 vaccination by race, income, and sexual behaviors. In general, the same pattern of significance is seen with greater proportions of women highly ranking male partner vaccination compared to not caring whether the male partner was vaccinated for demographic and sexual behavior characteristics. White women are the only racial group to show a significant difference in proportion of high rankings for ‘not caring’ whether the male partner is vaccinated: white women choosing HPV2 had a significantly greater proportion of high rankings than did the white women who received at least one dose of HPV4 (28% vs. 14%, p = 0.003). Similarly for women with more than two lifetime sexual partners, a greater proportion of women choosing HPV2 vaccination compared to having received at least one dose of HPV4 vaccine had high rankings for ‘not caring’ whether the male partner was vaccinated (32% vs. 15%, p = 0.008).

**Table 3:** Women's preference rankings for her male partner's HPV vaccination status by her demographic characteristics.

	Not vaccinated and choosing HPV2 for herself			Vaccinated with HPV4		
	Strongly agree/agree			Strongly agree/agree		
	n	%	p-value	n	%	p-value
White						
Male sexual partner vaccinated with HPV4	61/105	58.1%	<0.001	140/190	73.7%	<0.001
Don't care if male partner is vaccinated*	29/104	27.9%*		26/191	13.6%*	
Black						
Male sexual partner vaccinated with HPV4	31/34	91.2%	<0.001	36/43	83.7%	<0.001
Don't care if male partner is vaccinated	3/32	9.4%		3/41	7.3%	
Hispanic						
Male sexual partner vaccinated with HPV4	4/7	57.1%	0.035	6/10	60.0%	0.029
Don't care if male partner is vaccinated	0/7	0%		1/10	10.0%	
Asian						
Male sexual partner vaccinated with HPV4	16/22	72.7%	<0.001	11/16	68.8%	0.003
Don't care if male partner is vaccinated	3/21	14.3%		3/17	17.6%	
Income < $10,000/year						
Male sexual partner vaccinated with HPV4	49/67	73.1%	<0.001	75/104	72.1%	<0.001
Don't care if male partner is vaccinated	16/64	25.0%		15/104	14.4%	
Age at First Intercourse ≤15 years						
Male sexual partner vaccinated with HPV4	11/15	73.3%	0.030	33/46	71.7%	<0.001
Don't care if male partner is vaccinated	4/15	26.7%		5/46	10.9%	
Lifetime Number of Sexual Partners >2						
Male sexual partner vaccinated with HPV4	41/62	66.1%	<0.001	108/142	76.1%	<0.001
Don't care if male partner is vaccinated†	19/60	31.7%†		22/143	15.4%†	

*White women not already vaccinated and choosing HPV2 for herself had a significantly higher proportion of strongly agree/agree rankings regarding not caring if her male partner is vaccinated with HPV4 than do white vaccinated women (28% vs. 14%, p = 0.003).

† Women not already vaccinated and choosing HPV2 had a significantly higher proportion of strongly agree/agree rankings regarding not caring if her male partner is vaccinated with HPV4 than do vaccinated women when lifetime number of sexual partners exceeds 2 (32% vs. 15%, p = 0.008).

The preference for male vaccination had significantly higher preference rankings than not caring if the male is vaccinated by women who are condom and oral contraceptive users, as well as women with a history of an abnormal Pap test, HPV infection, colposcopy, or other STI's (Table 4). Few study participants had genital warts. As seen in Table 3, those women choosing HPV2 vaccine had a greater proportion of high preference rankings for not caring if the male is vaccinated compared to women having received at least one dose of HPV4 (31% vs. 17%, p = 0.007).

**Table 4:** Women's preference rankings for her male partner's vaccination status by her reproductive medical history.

	Not vaccinated and choosing HPV2 for herself			Vaccinated with HPV4		
	Strongly agree/agree			Strongly agree/agree		
	n	%	p-value	n	%	p-value
Condom Use (Currently/Ever)						
Male sexual partner vaccinated with HPV4	67/89	75.3%	<0.001	134/175	76.6%	<0.001
Don't care if male partner is vaccinated	17/87	19.5%		21/174	12.1%	
Oral Contraceptive Use (Currently/Ever)						
Male sexual partner vaccinated with HPV4	44/71	62.0%	<0.001	105/136	77.2%	<0.001
Don't care if male partner is vaccinated*	22/70	31.4%*		23/138	16.7%*	
History of having had an abnormal Pap test						
Male sexual partner vaccinated with HPV4	19/28	76.0%	<0.001	42/52	80.8%	<0.001
Don't care if male partner is vaccinated	3/23	12.0%		1/52	1.9%	
History of HPV infection						
Male sexual partner vaccinated with HPV4	6/8	75.0%	0.004	27/31	87.1%	<0.001
Don't care if male partner is vaccinated	0/8	0.0%		0/31	0.0%	
History of Colposcopy						
Male sexual partner vaccinated with HPV4	10/17	58.8%	0.004	28/30	93.3%	<0.001
Don't care if male partner is vaccinated	2/17	11.8%		1/30	3.3%	
History of Other STIs						
Male sexual partner vaccinated with HPV4	9/9	100.0%	<0.001	29/36	80.6%	<0.001
Don't care if male partner is vaccinated	0/9	0.0%		2/36	5.4%	
History of Genital Warts						
Male sexual partner vaccinated with HPV4	1/3	33.0%	NS	8/10	80.0%	<0.001
Don't care if male partner is vaccinated	0/3	0.0%		0/10	0.0%	

*Women not already vaccinated and choosing HPV2 for herself had a significantly higher proportion of high rankings regarding not caring if her male partner is vaccinated with HPV4 than do vaccinated women (31% vs. 17%, p = 0.007).

## Discussion

This work shows that women prefer their male partners to be vaccinated regardless of their choice of vaccine for themselves. This preference is pronounced when the woman has one or more perceived vulnerabilities, e.g. high number of lifetime sexual partners, a past history of HPV infection, abnormal Pap testing, colposcopy or another STI. No other study has reported on women's preferences for male HPV vaccination. One study has reported male preferences for cervical cancer screening every 3 years among female family members without any additional preference for HPV vaccination [21].

The cost-effectiveness models show that male HPV vaccination is only cost effective when several conditions are met. These include receiving all three doses on time, assuming lifetime duration of efficacy, vaccination occurs prior to HPV infection and vaccination coverage in the population of potential partnering females is less than 50% [22]–[27]. In addition, the more heterogeneous the sexual contact network of males and females, the less impact male vaccination has on reducing the population prevalence of HPV infection [28]. While this evidence dissuades male vaccination from a public health viewpoint, a recent mathematical model indicates that vaccinating females with HPV2 and males with HPV4 may maximize the prevention of invasive cervical cancer [29]. Our study has shown that a woman choosing HPV2 for herself expresses less value in having male partner vaccination than does a woman who has received at least one dose of HPV4. Reasons for this decreased value are speculative but may be related to the information provided that shows that 93% of CIN 3 from any HPV type are prevented by HPV2 vs. 43% by HPV4 [30] and this difference in protection was perceived to be significant enough that she was not as needy for male vaccination.

Other male HPV vaccination studies have focused on parental views about vaccinating sons, male attitudes about vaccine receipt, with some focus on men as male partners, and the provider recommendation for male HPV vaccination [31], all of which offer attitudinal support for male vaccination. The data show that few men (8.3%) have actually acted on this attitude [32] and fewer than 3% have completed the three dose series of HPV4 on time [33]. This study may offer influence within the social networking sphere for males to consider receiving HPV4 at the encouragement of their female friends.

### Limitations

This study was conducted on an urban US Midwest college campus among women 18–26 years of age, and as such may not be representative of the perspectives of young adolescent girls or their parents. We did not require the women who had been vaccinated to specify how many doses of vaccine they received.

We did not offer women the choice of no vaccination for herself and vaccination for her male partner; hence we do not know what proportion of women would choose to shift the burden of HPV infection prevention to the male partner. Likewise, we did not ask women their last menstrual period date or cycle regularity, as some sexual preferences for male behavior are linked to the reproductive cycle [34]. Nor did we ask about male partner concurrency. Furthermore, the preferences elicited here are based on intent and may not necessarily be realized.

## Conclusions

Women rank highly their preferences for male HPV vaccination. These choices are intensified if the woman has received at least one dose of HPV4 herself.

## References

[1] Alexander AB, Stupiansky NW, Ott MA, Herbenick D, Reece M, et al (2012) Parent-son decision-making about human papillomavirus vaccination: a qualitative analysis. BMC Pediatr 12: 192. 23241217 10.1186/1471-2431-12-192 PMC3547753

[2] Ogilvie GS, Remple VP, Marra F, McNeil SA, Naus M, et al (2008) Intention of parents to have male children vaccinated with the human papillomavirus vaccine. Sex Transm Infect 84: 318–23. 18445636 10.1136/sti.2007.029389

[3] Podolsky R, Cremer M, Atrio J, Hochman T, Arslan AA (2009) HPV vaccine acceptability by Latino parents: a comparison of U.S. and Salvadoran populations. J Pediatr Adolesc Gynecol 22(4): 205–15. 19646665 10.1016/j.jpag.2008.05.010

[4] Watts LA, Joseph N, Wallace M, Rauh-Hain JA, Muzikansky A, et al (2009) HPV vaccine: A comparison of attitudes and behavioral perspectives between Latino and non-Latino women. Gynecol Oncol 112: 577–82. 19150120 10.1016/j.ygyno.2008.12.010

[5] Riedesel JM, Rosenthal SL, Zimet GD, Bernstein DI, Huang B, et al (2005) Attitudes about human papillomavirus vaccine among family physicians. J Pediatr Adolesc Gynecol 18(6): 391–8. 16338604 10.1016/j.jpag.2005.09.004

[6] Daley MF, Liddon N, Crane LA, Beaty BL, Barrow J, et al (2006) A national survey of pediatrician knowledge and attitudes regarding human papillomavirus vaccination. Pediatrics 118: 2280–9. 17142510 10.1542/peds.2006-1946

[7] Hilton S, Patterson C, Smith E, Bedford H, Hunt K (2013) Teenagers' understandings of and attitudes towards vaccines and vaccine-preventable diseases: a qualitative study. Vaccine 31: 2543–50. 23602536 10.1016/j.vaccine.2013.04.023 PMC3679446

[8] Wong LP, Sam IC (2010) Ethnically diverse female university students' knowledge and attitudes toward human papillomavirus (HPV), HPV vaccination and cervical cancer. Eur J Obstet Gynecol Reprod Biol 148(1): 90–5. 19910102 10.1016/j.ejogrb.2009.10.002

[9] Licht AS, Murphy JM, Hyland AJ, Fix BV, Hawk LW, et al (2010) Is use of the human papillomavirus vaccine among female college students related to human papillomavirus knowledge and risk perception? Sex Transm Infect 86: 74–8. 19841004 10.1136/sti.2009.037705

[10] Gerend MA, Barley J (2009) Human papillomavirus vaccine acceptability among young adult men. Sex Transm Dis 36: 58–62. 18830138 10.1097/OLQ.0b013e31818606fc

[11] Reiter PL, Brewer NT, Smith JS (2010) Human papillomavirus knowledge and vaccine acceptability among a national sample of heterosexual men. Sex Transm Infect 86: 241–6. 19951936 10.1136/sti.2009.039065 PMC4028225

[12] Richmond MJ, Mermelstein RJ, Metzger A (2012) Heterogeneous Friendship Affiliation, Problem Behaviors, and Emotional Outcomes among High-Risk Adolescents. Prev Sci 13: 267–277. 22207411 10.1007/s11121-011-0261-2 PMC3354038

[13] Dishion TJ, Dodge KA (2005) Peer Contagion in Interventions for Children and Adolescents: Moving Towards an Understanding of the Ecology and Dynamics of Change. J Abnorm Child Psychol 33: 395–400. 15957566 10.1007/s10802-005-3579-z PMC2745625

[14] Salvy SJ, de la Haye K, Bowker JC, Hermans RC (2012) Influence of peers and friends on children's and adolescents' eating and activity behaviors. Physiol Behav 106: 369–78 10.1016/j.physbeh.2012.03.022 22480733 PMC3372499

[15] Leigh WA, Andrews JL (2002) The Reproductive Health of African American Adolescents: What We Know and What We Don't Know. Peer influence on sexual behavior: what we know about African American Teens. (Washington, DC: Joint Center for Political and Economic Studies). pp 1–11. Available: http://www.aecf.org/upload/publicationfiles/peer%20influence%20sexual%20behavior.pdf Accessed August 15, 2013.

[16] Rice E, Milburn NG, Monro W (2011) Social Networking Technology, Social Network Composition, and Reductions in Substance Use Among Homeless Adolescents. Prev Sci 12: 80–88. 21194011 10.1007/s11121-010-0191-4 PMC3040814

[17] Ali MM, Amialchuk A, Dwyer DS (2011) Social network effects in contraceptive behavior among adolescents. J Dev Behav Pediatr 32: 563–71 10.1097/DBP.0b013e318231cf03 21918469

[18] Harper DM, Vierthaler SL, Santee JA (2010) Review of HPV4. J Vaccines Vaccin (1) 107 doi:http://www.omicsonline.org/2157-7560/2157-7560-1-107.pdf. Accessed 15 August 2013 10.4172/2157-7560.1000107 PMC3690661 23805398

[19] Harper DM, Irons BB, Alexander NM, Comes JC, Smith MS, et al (2014) Quantifying the Decisional Satisfaction to Accept or Reject the Human Papillomavirus (HPV) Vaccine: A Preference for Cervical Cancer Prevention. PLoS One 9: e88493 10.1371/journal.pone.0088493 24551110 PMC3925140

[20] StatSoft, Inc(2010) STATISTICA (data analysis software system), version 12. www.statsoft.com.

[21] Oteng B, Marra F, Lynd LD, Ogilvie G, Patrick D, et al (2011) Evaluating societal preferences for human papillomavirus vaccine and cervical smear test screening programme. Sex Transm Infect 87: 52–7. 20956352 10.1136/sti.2009.041392

[22] Canfell K, Chesson H, Kulasingam SL, Berkhof J, Diaz M, et al (2012) Modeling preventative strategies against human papillomavirus-related disease in developed countries. Vaccine 30 Suppl 5 F157–67 10.1016/j.vaccine.2012.06.091 23199959 PMC3783354

[23] Smith MA, Lew JB, Walker RJ, Brotherton JM, Nickson C, et al (2011) The predicted impact of HPV vaccination on male infections and male HPV-related cancers in Australia. Vaccine 29: 9112–22. 21419773 10.1016/j.vaccine.2011.02.091

[24] Chesson HW, Ekwueme DU, Saraiya M, Dunne EF, Markowitz LE (2011) The cost-effectiveness of male HPV vaccination in the United States. Vaccine 29: 8443–50. 21816193 10.1016/j.vaccine.2011.07.096

[25] Marty R, Roze S, Bresse X, Largeron N, Smith-Palmer J (2013) Estimating the clinical benefits of vaccinating boys and girls against HPV-related diseases in Europe. BMC Cancer 13: 10. 23298365 10.1186/1471-2407-13-10 PMC3561184

[26] Kim JJ, Brisson M, Edmunds WJ, Goldie SJ (2008) Modeling cervical cancer prevention in developed countries. Vaccine 26 Suppl 10 K76–86 10.1016/j.vaccine.2008.06.009 18847560 PMC2769256

[27] Brisson M, van de Velde N, Franco EL, Drolet M, Boily MC (2011) Incremental impact of adding boys to current human papillomavirus vaccination programs: role of herd immunity. J Infect Dis 204: 372–6. 21742835 10.1093/infdis/jir285

[28] Bogaards JA, Kretzschmar M, Xiridou M, Meijer CJ, Berkhof J, et al (2011) Sex-specific immunization for sexually transmitted infections such as human papillomavirus: insights from mathematical models. PLoS Med 8: e1001147 10.1371/journal.pmed.1001147 22205887 PMC3243713

[29] Drolet M, Boily MC, Van de Velde N, Franco EL, Brisson M (2013) Vaccinating Girls and Boys with Different Human Papillomavirus Vaccines: Can It Optimise Population-Level Effectiveness? PLoS One 8: e67072. 23840589 10.1371/journal.pone.0067072 PMC3694081

[30] Heitmann E, Harper DM (2012) Prophylactic HPV Vaccines and Prevention of Cervical Intraepithelial Neoplasia. Ayhan A (Section Ed) Vulvar and Cervical Lesions and Prevention. Current Obstetrics and Gynecology Reports. 10.1007/s13669-012-0017-4.

[31] Zimet GD, Rosenthal SL (2010) HPV vaccine and males: issues and challenges. Gynecol Oncol 117(2 Suppl):S26–31. 10.1016/j.ygyno.2010.01.028 20129653

[32] Dorell C, Stokley S, Yankey D, Jeyarajah J, MacNeil J, et al (2012) Centers for Disease Control and Prevention. National and State Vaccination Coverage Among Adolescents Aged 13–17 Years — United States, 2011. MMWR 61: 671–677.22932301

[33] Shen-Gunther J, Shank JJ, Ta V (2011) Gardasil HPV vaccination: surveillance of vaccine usage and adherence in a military population. Gynecol Oncol 123: 272–7. 21864887 10.1016/j.ygyno.2011.07.094

[34] Gangestad SW, Simpson JA, Cousins AJ, Garver-Apgar CE, Christensen PN (2004) Women's preferences for male behavioral displays change across the menstrual cycle. Psychological Science 15: 203–207. 15016293 10.1111/j.0956-7976.2004.01503010.x

